# Volumetric brain correlates of gait associated with cognitive decline in community-dwelling older adults

**DOI:** 10.3389/fnagi.2023.1194986

**Published:** 2023-10-04

**Authors:** Victoria N. Poole, Shahram Oveisgharan, Lei Yu, Robert J. Dawe, Sue E. Leurgans, Shengwei Zhang, Konstantinos Arfanakis, Aron S. Buchman, David A. Bennett

**Affiliations:** ^1^Rush Alzheimer’s Disease Center, Rush University Medical Center, Chicago, IL, United States; ^2^Department of Orthopedic Surgery, Rush University Medical Center, Chicago, IL, United States; ^3^Department of Neurological Sciences, Rush University Medical Center, Chicago, IL, United States; ^4^Department of Diagnostic Radiology and Nuclear Medicine, Rush University Medical Center, Chicago, IL, United States; ^5^Department of Family and Preventive Medicine, Rush University Medical Center, Chicago, IL, United States

**Keywords:** older adults, gait speed, cognitive decline, attention, executive functioning, memory, MRI, brain volumes

## Abstract

**Objective:**

To determine the extent to which the regional brain volumes associated with slow gait speed can inform subsequent cognitive decline in older adults from the Rush Memory and Aging Project.

**Approach:**

We utilized deformation-based morphometry (DBM) in a whole-brain exploratory approach to identify the regional brain volumes associated with gait speed assessed over a short distance during an in-home assessment. We created deformation scores to summarize the gait-associated regions and entered the scores into a series of longitudinal mixed effects models to determine the extent to which deformation predicted change in cognition over time, controlling for associations between gait and cognition.

**Results:**

In 438 older adults (81 ± 7; 76% female), DBM revealed that slower gait speed was associated with smaller volumes across frontal white matter, temporal grey matter, and subcortical areas and larger volumes in the ventricles during the same testing cycle. When a subset was followed over multiple (5 ± 2) years, slower gait speed was also associated with annual declines in global cognition, executive functioning, and memory abilities. Several of the gait-related brain structures were associated with these declines in cognition; however, larger ventricles and smaller medial temporal lobe volumes proved most robust and attenuated the association between slow gait and cognitive decline.

**Conclusion:**

Regional brain volumes in the ventricles and temporal lobe associated with both slow gait speed and faster cognitive decline have potential to improve risk stratification for cognitive decline in older adults.

## 1. Introduction

As a volitional behavior made more complex by both internal and external demands, the once simple act of walking requires an increasingly diverse assortment of higher-order neural resources as we age (Heuninckx et al., 2008; Seidler et al., 2010). Since several of these resources extend from traditional motor control regions to those shared by cognitive functions, slow gait can be associated with underlying neurological dysfunction and may even serve as an early marker of neurodegeneration and impending cognitive decline (Grande et al., 2019; Lee et al., 2019). However, as a vital sign of general health and well-being (Fritz and Lusardi, 2009), gait speed is also a non-specific predictor of *several* adverse health-related outcomes in older age (Abellan van Kan et al., 2009). Therefore, to identify the slow-walking older adults at greatest risk for cognitive decline, it is important to further deconstruct gait speed into the mechanisms closer to this association.

Growing cross-sectional evidence suggest that slow gait and cognitive dysfunction in older adults free of overt neurological disease are linked by way of neurodegeneration and structural damage presenting as brain atrophy and white matter lesions (Amboni et al., 2013; Grande et al., 2019). However, only two population-based studies to date have probed the structural correlates of slow gait speed and linked them directly to future cognitive decline. Rosso et al. (2017) reported that smaller grey matter volumes in the right hippocampus mediated the association between gait slowing and incident cognitive impairment in nearly 200 community-dwelling older adults from the Health ABC Study. Tajimi et al. (2023) later reported that white matter hyperintensity (WMH) burden and smaller grey matter volumes in the hippocampus and insula mediated the association between maximum gait speed and incident dementia in over 1,000 older adults from the Hisayama Study. However, given that both studies were conducted in moderate-to-high functioning older adults, it remains unknown whether similar substrates link slow gait and cognitive decline in individuals with a lower range of motor abilities.

The current study uses deformation-based morphometry, an MR technique able to quantify individual differences in morphometry and volume throughout the brain (Ashburner et al., 1998), to identify the regional patterns of brain atrophy associated with slow gait. We then examined the extent to which these MR measures could predict subsequent decline in cognitive abilities above and beyond gait speed in over 400 community-dwelling older adults from the Rush Memory and Aging Project (Bennett et al., 2005).

## 2. Methods

### 2.1. Study design

Participants came from the Rush Memory and Aging Project (MAP), a longitudinal study of older adults living within retirement communities, subsidized housing facilities, or individual homes in and around the greater Chicagoland area (Bennett et al., 2012). Launched in 1997, MAP studies the factors contributing to cognitive and motor decline, risk of AD and other dementias, and loss of independence in older age. To be eligible for the study, participants were older, without known dementia at time of enrollment, and agreed to annual evaluations and blood draws, as well as donation of brain, spinal cord, nerve, and muscle at time of death. The biennial neuroimaging sub-study further requires that individuals not have any contraindication to MRI. All studies and protocols were approved by an Institutional Review Board of Rush University Medical Center. Written consent was obtained from all participants prior to enrollment in the study, as well as an Anatomic Gift Act.

The current analysis included dementia-free participants that completed a motor assessment, neuropsychological battery, and neuroimaging protocol over a one-year testing cycle. Analytic baseline was defined as the first visit with structural data obtained on a 3 T Siemens TIM Trio scanner.

### 2.2. Clinical evaluation

Annual clinical evaluations were conducted via structured in-home testing in the community setting. This assessment included a thorough interview of medical history, musculoskeletal pain, and recent symptoms of depression, as measured by a modified 10-item version of the Center for Epidemiologic Studies Depression scale (Kohout et al., 1993). Body mass index (BMI) was calculated from height and weight measurements. Demographic information, including date of birth, sex, self-identified race, and years of education, were recorded at the parent study baseline interview.

### 2.3. Cognitive assessment

Cognitive abilities were assessed via 21 tests, 19 of which were selected by neuropsychologists to inform on areas of cognition affected in aging and dementia (Wilson et al., 2002). Briefly, these tests were administered by research assistants certified using performance-based criteria. Data were collected on laptop computers with forms programmed in Blaise (Central Bureau of Statistics, Voorburg, Netherlands) and scored in SAS (SAS Institute Inc., Cary, NC, USA). To address skew and minimize floor and ceiling effects, raw test scores were converted to z-scores using the parent study baseline mean and standard deviation, then averaged together to yield a global cognitive composite score, as described in prior publications (Wilson et al., 2015). As secondary outcomes of interest, we also computed composites for three cognitive domains of interest: general attention (Stroop word reading, number comparisons, and digit span forward), executive functioning (Stroop color naming, category fluency, and digit ordering), and episodic memory (immediate and delayed story recall, word list recall, and recognition) abilities, which were previously shown to be differentially associated with gait in older age (Poole et al., 2022). Further psychometric information about individual tests and composites may be found in earlier publications (Wilson et al., 2002).

### 2.4. Gait speed assessment

To evaluate gait, participants were asked to walk at a self-selected pace across an 8-foot (i.e., 2.4 m) path. This distance was selected to accommodate limited testing space in participant homes and has been shown to be both reliable (Ostchega et al., 2000) and comparable to longer distances (Bohannon, 2008). Gait speed (m/s) was calculated using the average time of two trials, as measured by stopwatch.

### 2.5. Imaging

During the neuroimaging visit, participants were imaged on a Siemens 3 T Magnetom Trio MRI (Siemens Medical Solutions, Erlangen, Germany) with a high-resolution T1-weighted MPRAGE sequence (TR/TE/TI = 2300/2.98/900 ms, acquisition voxel dimensions 1x1x1 mm^3^, PAT 2, flip angle 9°, scan time 330 s). These data were later processed according to standard deformation-based morphometry (DBM) procedures using the Advanced Normalization Tools (ANTs) toolkit (Avants et al., 2009; Tustison et al., 2021). Specifically, T1 images were corrected for bias field inhomogeneity (Tustison et al., 2010), skull-stripped (Isensee et al., 2019), and non-linearly registered to the T1-weighted template of the MIITRA atlas (Ridwan et al., 2021). Then, voxel-wise maps of the Jacobian determinant were generated from the transformation matrices used in subject-to-template registration, log transformed, and smoothed with a 4-mm FWHM Gaussian kernel. A higher value in these deformation maps indicated large volumes that had to be contracted to fit the template, while a lower value indicated smaller volumes that had to be expanded to the template. T2-weighted FLAIR images (TR/TE/TI = 9000/150/249 ms, acquisition voxel dimensions 0.9 × 0.9 × 0.9 mm^3^, PAT 2, flip angle 150°, scan time 162 s) were also collected to assess white matter hyperintensity (WMH) burden, calculated as the WMH lesion volume by BIANCA (Griffanti et al., 2016) and expressed as a percentage of the total brain intracranial volume.

### 2.6. Statistical analysis

We first examined the associations between gait speed and participant characteristics using Spearman’s rank correlations and Mann–Whitney U tests. We then examined the associations between gait speed and regional deformation by conducting voxel-wise linear regression analyses using FSL’s randomize (Winkler et al., 2014) with 500 permutations, threshold-free cluster enhancement, and family-wise error rate (FWER) correction. Contiguous voxels exceeding FWER *p* < 0.005 with at least 0.54 mL volume (i.e., 540 1x1x1mm^3^ voxels) were used to generate gait-related subject-level cluster medians. Next, to reduce dimensionality and recognize covariance across the gait-related brain volumes that were not spatially contiguous, we conducted principal component analyses (PCA) on the z-scored estimates and created deformation structure scores guided by the PCA’s rotated factor pattern. These scores represented overall patterns of brain volumes or reserve across the identified gait-related regions. To determine the optimal number of principal components, we first examined the number of factors corresponding to eigenvalues of the correlation matrix greater than 1. We then performed a varimax (e.g., orthogonal) rotation and were guided by the sign and effect size (cut-off = 0.5) to interpret factor loadings. However, when we further examined an additional factor with eigenvalue = 0.92, we identified groupings that were more interpretable and consistent with anatomical pairings and literature. We then confirmed their robust associations with gait speed by performing linear regression models controlling for three sets of relevant covariates: an initial “core” set of participant demographics, i.e., age, sex, self-reported race, and level of education; next, a set of clinical characteristics at the time of visit, i.e., body mass index, self-reported joint and/or lower extremity pain, and depressive symptoms; and finally, a set of cerebrovascular covariates, i.e., self-reported history of stroke and MRI-derived WMH burden.

To model cognitive abilities over time, we ran an initial linear mixed-effects model of the global cognitive composite with random effects allowing for person-specific levels and rates of decline, adjusted for “core” covariates: age, sex, race, education, BMI, and their interactions with the time since analytic baseline. These covariates were selected as notable sources of variation in cognitive decline that may also confound its associations with gait speed and regional brain volumes (Arvanitakis et al., 2018; Dekkers et al., 2019; Mantel et al., 2019). We then added gait speed and its interaction with time to examine gait associations with level of cognition at baseline and with rate of cognitive decline, respectively. To determine the extent to which the gait-related brain structures were associated with cognition, the core mixed effects models were also fitted for each of the PC deformation scores, each one separately, then together in a single model. The latter was used to determine the most robust structural associations with cognitive decline and select the scores to model jointly with gait speed. Statistics reported include the reduction in variation of the intercept/slope (delta-s^2), with larger percentages explained by the additional covariates. Finally, as a secondary analysis, we repeated these steps to model changes in attention, executive functioning, and memory abilities. Statistical programming was done in SAS v.9.4 for Linux (SAS Institute Inc., Cary, NC) and R software.

## 3. Results

### 3.1. Participant characteristics

At the time of analysis, 438 participants had gait testing and structural MRI data of sufficient quality on a single scanner during the same testing cycle. As shown in Table 1, participants were 81 ± 7 (mean ± SD) years of age, 76% female, 95% white, completed 16 ± 3 years of education, and walked at an average speed of 0.5 ± 0.2 m/s. The average MMSE score was 28 ± 1.5 and the global cognitive composite score was 0.3 standard units, indicating the group scored approximately 0.3 SD higher on the cognitive battery than the parent cohort at baseline. Of the 438, 422 completed at least two follow-up cognitive assessments (follow-up time mean ± SD = 5 ± 2 years, range = 2–10 years) and declined at a rate of 0.09 units on the 19-test battery per year.

**Table 1:** Participant characteristics.

Characteristic	Data (*n* = 438)
Age, mean (SD), y	81 (7.3)
Women, no. (%)	333 (76)
Non-Latino white, no. (%)	418 (95)
Educational level, mean (SD), y	15.9 (3)
Body mass index, mean (SD)	27.4 (5.2)
Self-reported pain in joints, no. (%)	186 (42)
Pain in lower extremities, no. (%)	137 (31)
Depressive symptoms, mean (SD)	0.7 (1.3)
History of stroke, no. (%)	33 (7.6)
Hypertension, no. (%)	236 (54)
WMH burden, mean (SD), %ICV	1.2 (2.1)
Gait speed, mean (SD), m/s	0.5 (0.2)
MMSE score, mean (SD)	28 (1.5)
Global cognitive composite score, mean (SD)	0.3 (0.5)
Global cognitive composite slope (*n* = 422), points per year, mean (SD)	−0.09 (0.1)

MMSE, mini-mental state examination; WMH, white matter hyperintensity; ICV, intracranial volume.

### 3.2. Gait speed and deformation-based morphometry

**Figure 1:**
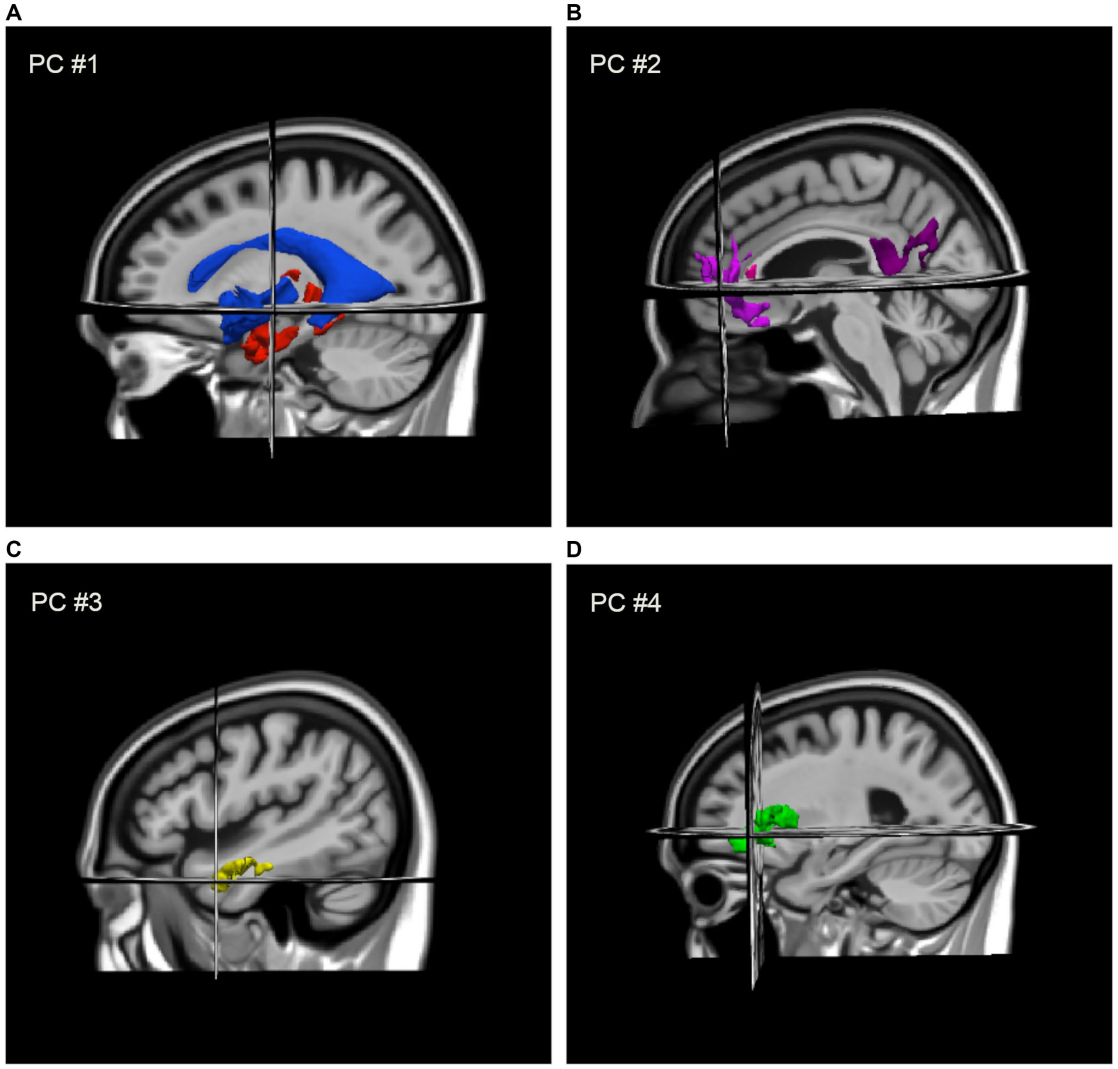
Regional deformation patterns associated with gait speed, as assigned to four PCA factor-derived deformation scores: **(A)** contraction (i.e., larger volumes) in the medial temporal lobe and thalamus with expansion (i.e., smaller volume) of the ventricles (i.e., PC #1) to the MIITRA template; **(B)** contraction across the left anterior cingulate, right middle frontal gyrus and insula, and the genu and splenium of the CC (i.e., PC #2); **(C)** contraction across the left and right anterolateral temporal lobes (i.e., PC #3); and **(D)** contraction across the left and right insula (i.e., PC #4).

DBM revealed positive associations between gait speed and deformation across several brain regions including frontal white matter, temporal grey matter, limbic, and other subcortical areas, such that smaller volumes in these regions were associated with slower gait. Negative associations with gait speed were also observed with deformation across the ventricles and CSF spaces. PCA revealed four components (i.e., patterns of deformation), which accounted for 68% of variance across 13 structures: contraction in the medial temporal lobe and expansion of the ventricles (i.e., PC #1, 43%); contraction across the left anterior cingulate, right middle frontal gyrus and insula, and the corpus callosum (i.e., PC #2, 10%); contraction across the left and right anterolateral temporal gyrus (i.e., PC #3, 8%); and contraction across the left and right insula (i.e., PC #4, 7%). These components, shown in Figure 1, were coded in such a way that higher scores indicated larger tissue volumes and smaller ventricles. Lower PC scores were associated with slower gait speed and the four scores collectively accounted for an additional 10% of variance in gait speed beyond participant characteristics.

Additional details on the individual components are provided in Supplementary Tables S2, S3; a complete map of all DBM associations with gait speed without cluster thresholding is provided in Supplementary Figure S1.

### 3.3. Gait speed and cognition

As expected, linear mixed-effects models indicated that slower gait speed was associated with lower global cognition at the time of testing (*b* = 0.12, *p* < 0.001, 95%CI [0.07, 0.16]; change in explained variance: Δs^2^ = 6%) and a faster rate of cognitive decline (*b* = 0.013, *p* = 0.035, 95%CI [9E-4, 0.025]; Δs^2^ = 2%; Supplementary Figure S3), after adjusting for age, sex, race, education, and BMI. When we repeated these models for individual cognitive domains, slower gait was associated with poorer performance across all three domains (*p*’s < 0.001) and annual declines in executive function (*b* = 0.021, *p* = 0.005, 95%CI [6.4E-3, 0.035]; Δs^2^ = 3%) and memory (*b* = 0.015, *p* = 0.05, 95%CI [4.1E-6, 0.03]; Δs^2^ < 1%). Gait speed was not associated with the annual rate of change in the attentional composite (*b* = −0.003, *p* = 0.65, 95%CI [−0.016, 0.01]; Δs^2^ < 1%).

### 3.4. DBM and cognition

To determine whether gait-related brain structures were also associated with subsequent cognitive decline, we then entered the four deformation scores into separate and joint models of cognition. When modeled separately, three of the four scores were associated with the annual rate of change in global cognition, such that lower scores were associated with faster decline (*p*’s < 0.008). When modeled together, only PC #1 (*b* = 0.053, *p* < 0.001, 95%CI [0.032, 0.073]) and PC #3 (*b* = 0.019, *p* = 0.02, 95%CI [1.8E-3, 0.033]) remained significant predictors of decline (Table 2; Supplementary Figure S4). Together, these two scores accounted for an additional 13% of variance in cognitive decline beyond demographic characteristics. PC #1 was also most strongly associated with the annual change in each of the cognitive domains, as shown in Supplementary Table S5.

**Table 2:** Mixed effects models of global cognition: associations of gait-related deformation PC scores with cognitive level and change, modeled separately and jointly.

	Association with cognitive level				Association with cognitive change			
	Separate		Joint		Separate		Joint	
PC score	*b* (95% CI)	*p*	*b* (95% CI)	*p*	*b* (95% CI)	*p*	*b* (95% CI)	*p*
1	0.172 (0.106, 0.239)	<0.001	0.114 (0.037, 0.192)	0.004	0.057 (0.039, 0.075)	<0.001	0.053 (0.032, 0.073)	<0.001
2	0.137 (0.069, 0.205)	<0.001	0.049 (−0.042, 0.14)	0.29	0.026 (0.006, 0.044)	0.008	0.005 (−0.018, 0.029)	0.66
3	0.113 (0.058, 0.168)	<0.001	0.077 (0.021, 0.134)	<0.01	0.030 (0.015, 0.045)	<0.001	0.019 (0.004, 0.034)	0.02
4	0.104 (0.046, 0.162)	<0.001	0.021 (−0.053, 0.094)	0.58	0.013 (−0.003, 0.029)	0.12	−0.011 (−0.031, 0.008)	0.26

All models were adjusted for age, sex, race, education, BMI, and their interactions with time.

### 3.5. Gait speed, DBM, and cognition

Finally, to determine the extent to which the structure scores were associated with cognitive decline above and beyond slow gait speed, we included PC #1 and PC #3 together with gait speed in the same covariate-adjusted model (Table 3). In this model, both PC #1 (*b* = 0.050, *p* < 0.001, 95%CI [0.032, 0.069]) and PC #3 (*b* = 0.017, *p* = 0.04, 95%CI [0.001, 0.032]) remained significant predictors of change in global cognition; however, gait speed was no longer significant (*b* = 0.004, *p* = 0.51, 95%CI [−7.8E-3, 0.0157]). This combined model accounted for 11% more variance in the rate of change in global cognition than gait speed alone. Similar patterns of association were also observed for annual rates of change in executive function and memory (Supplementary Tables S6, S7). Of note, all associations with cognitive decline remained after further adjustment for white matter hyperintensity burden, an imaging marker of cerebrovascular damage also linked to compromised cognition and motor function in older adults (Soumaré et al., 2009; Arvanitakis et al., 2016; Boyle et al., 2016) (data not shown).

**Table 3:** Mixed effects models of global cognition: associations between gait-speed and gait-related deformation scores with cognitive level and change, modeled separately, and together.

	Cognitive level b, *p-val,* (95%CI)			Cognitive change b, *p-val,* (95%CI)		
	Model 1	Model 2	Model 3	Model 1	Model 2	Model 3
Gait speed	0.118, *p* < 0.001 (0.073, 0.164)	–	0.092, *p* < 0.001 (0.046, 0.138)	0.013, *p* = 0.035 (9E−4, 0.025)	–	0.004*, p* = 0.51 (−0.008, 0.016)
PC 1	–	0.146, *p* < 0.001 (0.077, 0.214)	0.133, *p* < 0.001 (0.066, 0.201)	–	0.051, *p* < 0.001 (0.032, 0.07)	0.050, *p* < 0.001 (0.032, 0.069)
PC 3	–	0.081, *p* = 0.005 (0.024, 0.137)	0.054*, p* = 0.06 (−0.003, 0.111)	–	0.018, *p* = 0.02 (2.62E−3, 0.033)	0.017, *p* = 0.04 (0.001, 0.032)
Explained variance	6%	8%	12%	2%	13%	13%

All models were adjusted for age, sex, race, education, BMI, and their interactions with time.

## 4. Discussion

The current study used DBM to investigate the gait-related brain structures that were associated with cognitive decline in over 400 older adult participants from the Rush Memory and Aging Project. We first used this technique to identify several brain structures associated with slow gait, including the frontal white matter, temporal grey matter, subcortical regions, and the ventricles. We then observed that of the regions also associated with cognition, limbic, ventricular, and temporal lobe volumes proved most robust and accounted for the relationship between slow gait and accelerated cognitive decline. These findings suggest that while gait speed offers insight into cognitive outcomes, individual differences in volumes across these regions are more strongly associated with decline and should be considered when assessing risk.

It has been firmly established that faster walking can be linked to better executive control, attention, language, visuospatial abilities, and overall cognitive functioning (Morris et al., 2016), while slower walking can indicate poorer function and higher risk for cognitive impairment and dementia in older age (Dumurgier et al., 2017; Grande et al., 2019). In the current study, we observed that slower walking speed was associated with lower global cognitive abilities and poorer performance across three assessed cognitive domains. Positive associations with executive functioning, attention, and memory were anticipated and provided evidence that slow walking may indicate neurocognitive dysfunction in this cohort (Yogev-Seligmann et al., 2008). Importantly, slower gait speed at analytic baseline was also associated with faster declines in executive functioning, memory, and global cognitive abilities. Collectively, these findings agreed well with the proposed “Motoric Cognitive Risk” syndrome (Meiner et al., 2020), where slow walking speed may be used as a complementary risk factor for dementia when present with memory difficulties.

Regarding the volumetric correlates of gait speed, we report several brain structures that are consistent with other studies though conducted in an exploratory voxel-wise manner across both tissues and non-tissue structures of the brain. Specifically, we observed a set of grey matter volumes in frontal cognitive and motor regions (Blumen et al., 2018), limbic and subcortical regions like the hippocampus and thalamus (Callisaya et al., 2014; Ezzati et al., 2015), white matter in frontal regions and corpus callosum (Srikanth et al., 2010; Poole et al., 2018), and ventricular dilation (Palm et al., 2009) linked to individual differences in gait speed in our sample. When we conducted a PCA to reduce dimensionality and identify networks of structures, we observed four distinct patterns of deformation: smaller medial temporal and subcortical structures paired with enlarged ventricles (PC #1), smaller volumes across white matter supporting the frontoparietal and salience networks (PC #2), smaller bilateral volumes in the anterolateral temporal cortex (PC #3), and smaller volumes in the insular cortex (PC #4).

Interestingly, although several of the structures are also known to support cognition and were associated with cognitive abilities at the time of testing, PC #1 drove associations with cognitive decline in our cohort. This PC captured the most variance in our sample and indicated substantial individual differences in brain volume (i.e., reserve) across limbic regions that were inversely related to ventricular volumes. While the hippocampus was amongst the included regions (Rosso et al., 2017; Tajimi et al., 2023), this overall pattern was consistent with coupled limbic brain atrophy and ventriculomegaly observed in AD-related neurodegeneration (Tang et al., 2014). Our subsequent analyses suggest the degree to which an individual exhibited this pattern of atrophy explained the link between slow gait speed at baseline and later cognitive decline, particularly in executive functioning abilities. PC #3 offered a separate, albeit less significant pattern of cortical brain reserve in the temporal lobe linking gait and cognition. Taken together, these studies not only suggest the limbic and temporal lobes as common neural substrates between gait speed and cognition, but that gait speed is linked to subsequent cognitive decline in high-risk older adults largely to the extent that it is associated with underlying atrophy and pathology in these regions (Persson et al., 2006). Moreover, given that our findings suggest that measuring the reserve capacity (i.e., volume) of these regions offers far more insight towards cognitive decline than gait speed alone (13% vs. 2% variance explained for deformation-only and gait-only models), future work should confirm that the MR measurement of these volumes can improve risk stratification and recommendations for slow-walking older adults.

This study has several strengths and limitations that are worthy of discussion. First, we investigated the volumetric substrates of gait in a well-characterized and relatively large cohort of older adults at risk for cognitive decline. To facilitate ease of participation and eliminate some volunteer bias, we collected motor and cognitive data within the community, often in participants’ homes. As a result, our sample is likely less healthy but more representative of the older adult population than those normally assessed within the laboratory. We also asked participants to walk over a shorter distance than is typically used by other research teams, to accommodate participants’ homes. Our findings may be particularly noteworthy because of compelling evidence that the largest associations between cognitive performance and walking speed in less healthy older adults are observed over shorter walking distances (Pasma et al., 2014). However, our cohort lacked racial diversity and only featured ambulatory participants able to complete both MRI and gait protocols during the same testing cycle. Future work should determine the degree to which neuroimaging may complement slow gait as a risk factor for cognitive decline in a more diverse cohort. Finally, given that several person-specific mechanisms of cognitive decline are likely to exist (Boyle et al., 2018; Buchman et al., 2020), more advanced statistical approaches that incorporate multiple streams of data may better identify at-risk individuals.

In conclusion, our data show that lower brain reserve (i.e., atrophy) in limbic, periventricular, and temporal lobes are not only linked to slower gait but serve as superior predictors of subsequent cognitive decline in a cohort of community-dwelling older adults with mixed motor abilities. These findings support the use of quantitative MRI as a critical consideration when assessing older adults at risk for cognitive decline.

## Data availability statement

The datasets presented in this study can be found in online repositories. The names of the repository/repositories and accession number(s) can be found at: RADC Research Resource Sharing Hub (www.radc.rush.edu).

## Ethics statement

The studies involving humans were approved by an Institutional Review Board of Rush University Medical Center. The studies were conducted in accordance with the local legislation and institutional requirements. The participants provided their written informed consent to participate in this study.

## Author contributions

VP, AB, and DB conceived of the study, participated in its design, and acquired funding. KA, SZ, and VP acquired, processed, and/or analyzed neuroimaging data. SL, LY, and VP led the statistical analysis. VP wrote the first draft of the manuscript. SO and RD contributed to the interpretation of results. All authors contributed to the article and approved the submitted version.
