# Supplementary material for: Volumetric brain correlates of gait associated with cognitive decline in community-dwelling older adults
Source: Front Aging Neurosci. 2023 Oct 4;15:1194986. doi: 10.3389/fnagi.2023.1194986 (PMC10582745; doi:10.3389/fnagi.2023.1194986)
Supplement: Supplementary file 1 [file Data_Sheet_1.pdf]

Supplementary Material

Volumetric Brain Correlates of Gait Associated with Cognitive Decline in Community-dwelling Older Adults

Victoria N. Poole, Shahram Oveisgharan, Lei Yu, Robert J. Dawe, Sue E. Leurgans, Shengwei Zhang, Konstantinos Arfanakis, Aron S. Buchman, David A. Bennett

***Correspondence:** Victoria Poole: Victoria_Poole@rush.edu

Supplementary Table 1. Comparison of study sample and overall MAP participant characteristics at time of enrollment.

**Characteristic**	**MRI Subsample**	**Overall MAP**	**Included vs. Excluded Diff *p***
N	438	2275	438 vs. 1837
Age, mean (SD), y	79 (7.1)	80 (7.6)	<.001
Women, No. (%)	333 (76)	1341 (73)	.28
Non-Latino White, No. (%)	418 (95)	1602 (87)	<.001
Educational level, mean (SD), y	15.9 (3)	14.9 (3)	<.001
Body mass index, mean (SD)	27.4 (5.2)	27 (5.4)	.70
Self-reported Pain in joints, No. (%)	188 (43)	968 (43)	.91
Pain in lower extremities, No. (%)	144 (33)	771 (34)	.60
Depressive symptoms, mean (SD)	0.76 (1.3)	1.16 (1.7)	<.001
History of stroke, No. (%)	27 (6)	191 (8)	.07
Hypertension, No. (%)	213 (49)	1205 (53)	.049
Gait speed, mean (SD), m/s	0.59 (0.2)	0.59 (0.2)	.77
MMSE score, mean (SD)	28 (1.4)	27 (3.4)	<.001
Global cognitive composite score, mean (SD)	0.27 (0.5)	0 (0.7)	<.001

Supplementary Figure 1. All linear associations of regional brain volumes with gait speed surviving FWER correction at *p*=0.005, without cluster thresholding. Results are displayed using a heat colormap on the MIITRA template. Hot and cool colors (i.e., reds and blues) indicate the magnitude of positive and negative associations, respectively.

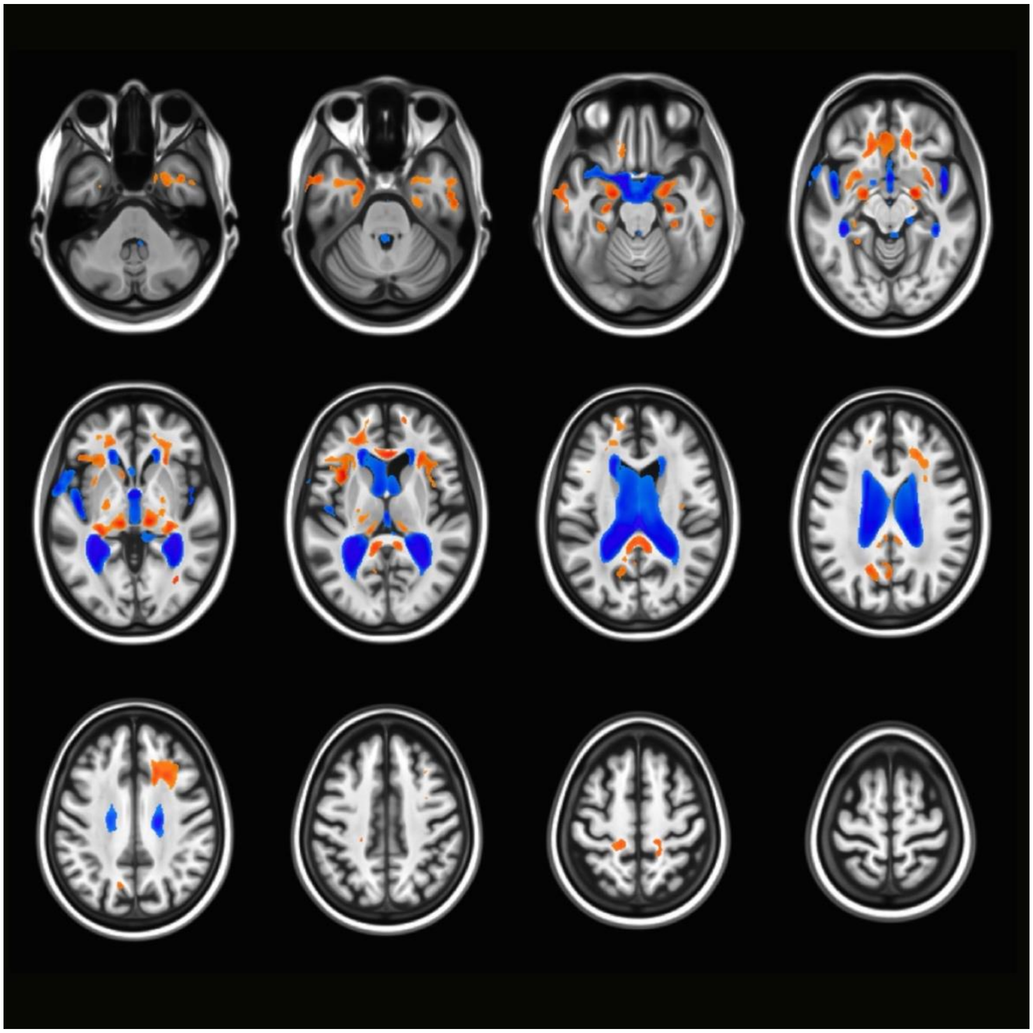

Supplementary Table 2. Regional deformations associated with gait speed (FWER *p*<.005, k>540).

			MIITRA Coordinates							
**PC**	**Cluster**		**Center of Mass**				**Peak Intensity**			
#	Voxels	Location	Mean	L-R	P-A	I-S	Max	L-R	P-A	I-S
**(-) 1**	71374	Ventricles & CSF	-3.976	4.2	19.8	11.5	-6.538	34	40	-1
**1**	6983	Left MTL (including the hippocampus, parahippocampal gyrus, and amygdala) and Thalamus (THA)	3.347	22.1	15.2	-7.1	5.323	27	30	0
**1**	5712	Right MTL and THA	3.372	-21	14	-9	6.078	-18	8	-7
**2**	4289	Splenium of Corpus Callosum (CC) & Left Posterior Cingulate	3.580	3.7	48.7	19.5	4.909	-8	45	14
**2**	3829	Left Anterior Cingulate	3.227	15.1	-33.9	1.9	4.597	4	-27	-8
**2**	3631	R Middle Frontal Gyrus (MFG)	3.248	-23.5	-18.9	31.6	4.506	-24	-15	34
**2/4**	3105	R Insula	3.306	-23.7	-23.9	4.2	4.847	-26	-23	9
**4**	2980	L Insula Lobe	3.320	32	-17	8.8	5.951	32	-12	12
**3**	1591	R Inferior Temporal Gyrus (ITG)	3.180	-51.2	14.3	-19.9	4.501	-52	16	-21
**3**	1437	L Middle/Inferior Temporal Gyrus	3.540	55.9	5.5	-18.4	4.551	59	-1	-24
	1345	Right Insula CSF Cavity	-4.354	-41.9	-0.6	-4.6	-5.862	-42	2	-5
**2**	763	Genu of CC	3.862	-0.6	-25.9	9.3	5.467	-3	-26	8
**1**	548	L Caudate Head	3.621	7.7	-7	-2.1	4.618	6	-6	-2

Supplementary Figure 2. Scatter plot and line of best fit showing the linear relationship between gait speed and age at analytic baseline, such that *gait speed* = 1 + 0.006 * *age in years* (p<.001).

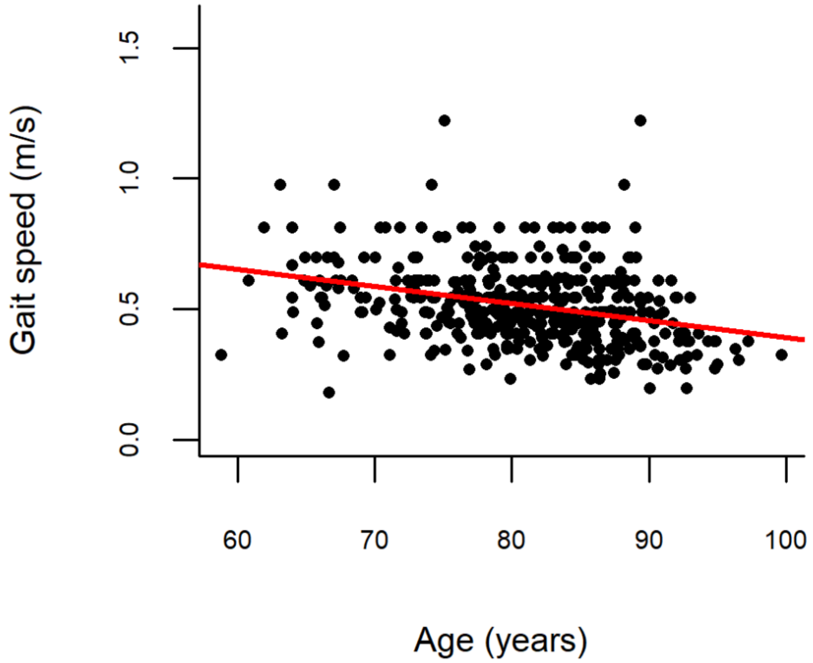

Supplementary Table 3. Linear associations between gait speed and regional deformation, according to three levels of adjustment. Model 1 (i.e., "Core") covariates: age, sex, self-reported race, level of education; Model 2: Core + body mass index, self-reported joint and/or lower extremity pain, and depressive symptoms; Model 3: Core + self-reported history of stroke and MRI-derived white matter hyperintensity burden.

		**Associations with Gait Speed**					
**Principal Component**		**Model 1**		**Model 2**		**Model 3**	
#	Location	b	*p*	b	*p*	b	*p*
1	L/R MTL + THA – Ventricles	.036	<.001	.036	<.001	.032	.002
2	L AC, R MFG, R Insula, g/s CC	.047	<.001	.045	<.001	.041	<.001
3	L/R ITG	.048	<.001	.046	<.001	.046	<.001
4	L/R Insula	.039	<.001	.038	<.001	.036	<.001

Supplementary Figure 3. (A) Plots showing cognitive function over time for a random sample of 50 participants. Each line is a single participant. (B) Predicted paths of global cognitive decline in a typical participant (i.e., 81-year-old white female with 17 years of education and a BMI of 27) with slower (10th percentile; dashed line), normal (50th percentile, solid line), vs. faster (90th percentile; dotted line) gait speed at time of testing.

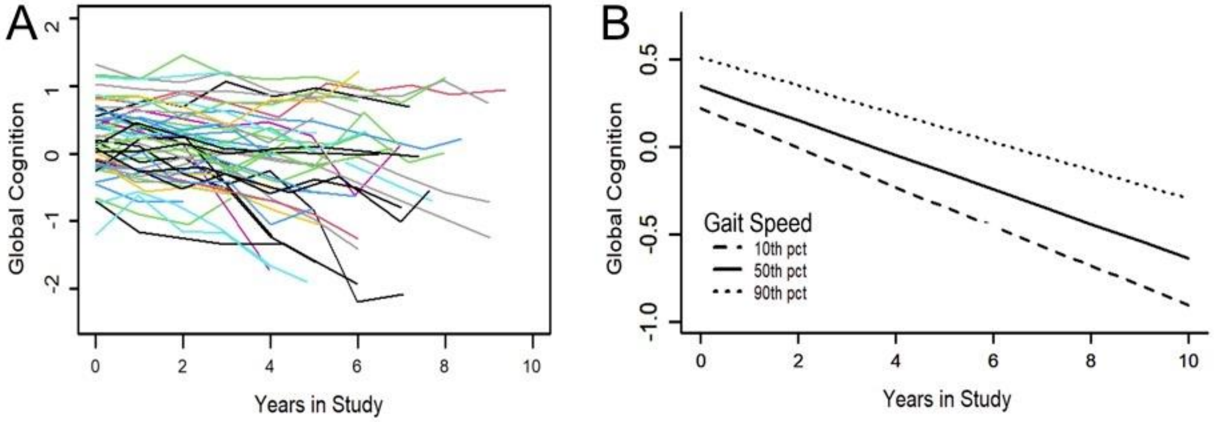

Supplementary Figure 4. Relation of gait-related deformation to trajectories of global cognition. Predicted paths of global cognitive decline in a typical participant (i.e., white 81-year-old female with 17 years of education and a BMI of 27) with high (90th percentile; dotted line), typical (50th percentile; solid line), and low (10th percentile; dashed line) deformation scores for (A) PC #1 and (B) PC #3, when these structures are modeled separately.

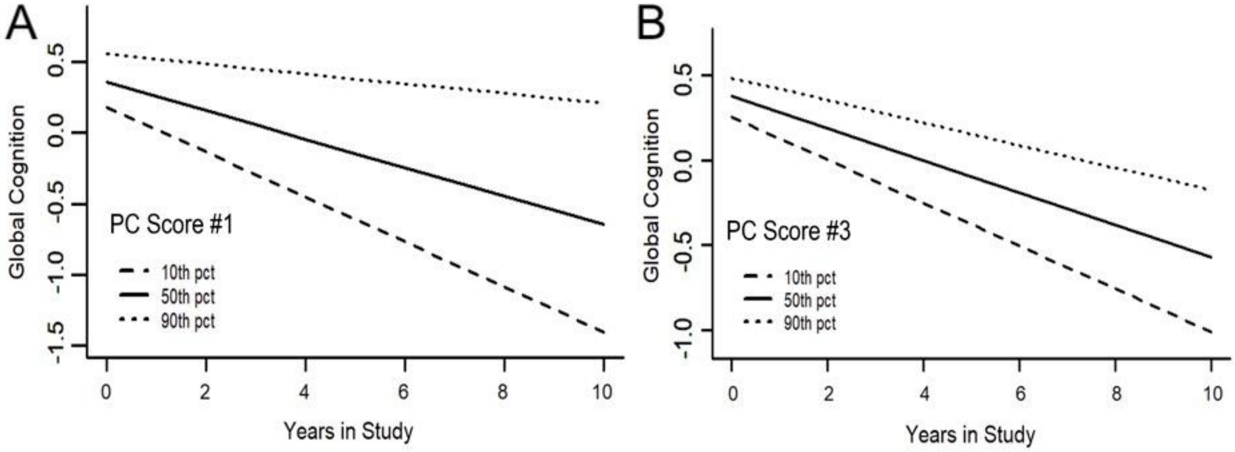

Supplementary Table 4. Model fit statistics for mixed effects models of global cognition.

Model	AIC	BIC	Slope Variance	Variance Explained (%)
Core only	1773	1789	1.10E-02	REF
Gait speed	1759	1775	1.08E-02	1.8
Deformation-based models				
PC #1	1726	1742	9.60E-03	12.7
PC #2	1764	1781	1.08E-02	1.8
PC #3	1757	1774	1.04E-02	5.5
PC #4	1772	1788	1.10E-02	0
PC #1-4	1746	1762	9.41E-03	14.5
**PC #1+3**	**1726**	**1743**	**9.60E-03**	**12.7**
Integrated model				
Gait speed, PC #1,3	1725	1741	9.60E-03	12.7

Supplementary Table 5. Mixed effects models of cognitive domains: Associations with gait-related deformation scores, as guided by PCA factor loadings, when modeling cognitive abilities separately and together. All models were adjusted for age, sex, race, education, BMI, and their interactions with time.

**Cognitive Composite**	**Deformation Score**	**Separate**				**Joint**			
		Level est. (95%CI)	*p*	Change est. (95%CI)	*p*	Level est. (95%CI)	*p*	Change est. (95%CI)	*p*
Executive Function	1	0.177 (0.088, 0.267)	<.001	0.058 (0.036, 0.081)	<.001	0.120 (0.015, 0.225)	.03	0.045 (0.019, 0.071)	<.001
	2	0.141 (0.05, 0.232)	.003	0.036 (0.013, 0.059)	.002	0.056 (-0.067, 0.178)	.37	0.012 (-0.018, 0.042)	.42
	3	0.112 (0.038, 0.186)	.003	0.035 (0.016, 0.053)	<.001	0.075 (-0.001, 0.151)	.05	0.023 (0.004, 0.042)	.02
	4	0.102 (0.025, 0.179)	.009	0.024 (0.004, 0.043)	.02	0.014 (-0.086, 0.113)	.78	-0.002 (-0.027, 0.022)	.86
Attention	1	0.087 (-0.01, 0.184)	.08	0.030 (0.009, 0.051)	.005	0.013 (-0.101, 0.127)	.82	.032 (0.008, 0.056)	.01
	2	0.110 (0.012, 0.208)	.03	.001 (-0.02, 0.022)	.91	0.053 (-0.08, 0.185)	.44	-0.011 (-0.039, 0.017)	.45
	3	0.111 (0.032, 0.19)	.006	.019 (0.002, 0.036)	.03	0.095 (0.012, 0.177)	.02	0.013 (-0.004, 0.031)	.14
	4	0.093 (0.011, 0.176)	.03	0.000 (-0.018, 0.019)	.96	0.039 (-0.069, 0.147)	.48	-0.006 (-0.029, 0.018)	.63
Memory	1	0.217 (0.13, 0.305)	<.001	0.078 (0.056, 0.101)	<.001	0.163 (0.061, 0.266)	.002	0.081 (0.055, 0.108)	<.001
	2	0.159 (0.068, 0.249)	<.001	0.024 (5.1E-4, 0.048)	<.05	0.069 (-0.051 0.19)	.26	-0.001 (-0.031, 0.03)	.97
	3	0.128 (0.055, 0.201)	<.001	.032 (0.013, 0.051)	.001	0.085 (0.01, 0.16)	.03	0.016 (-0.003, 0.035)	.11
	4	0.102 (0.026, 0.178)	.009	0.006 (-0.014, 0.027)	.55	-0.011 (-0.108, 0.086)	.82	-0.022 (-0.047, 0.002)	.08

Supplementary Table 6. Mixed effects model of executive function: Associations between gait-speed and gait-related deformation structures with cognitive level and change, modeled separately and together. All models were adjusted for age, sex, race, education, BMI, and their interactions with time.

	Level est., *p-val* (95%CI)			Change est., *p-val* (95%CI)		
	Model 1	Model 2	Model3	Model 1	Model 2	Model 3
Gait speed	0.123, *p*<.001 (0.063, 0.183)	-	0.096 *p*=.002 (0.034, 0.159)	0.021, *p*=.005 (0.006, 0.035)	-	0.012, *p*=.11 (-0.003, 0.026)
PC #1	-	0.152, *p*=.001 (0.059, 0.244)	0.139, *p*=.003 (0.047, 0.231)	-	0.05, *p*<.001 (0.026, 0.073)	0.048, *p*<.001 (0.025, 0.072)
PC #3	-	0.078, *p*=.04 (0.002, 0.154)	0.05, *p*=.20 (-0.027, 0.127)	-	0.023 *p*=.02 (0.004, 0.041)	0.019, *p*=.051 (-7E-5, 0.038)
Explained variance	4%	5%	7%	3%	10%	12%

Supplementary Table 7. Mixed effects model of episodic memory: Associations between gait-speed and gait-related deformation structures with cognitive level and change, modeled separately and together. All models were adjusted for age, sex, race, education, BMI, and their interactions with time.

	Level est., *p-val* (95%CI)			Change est., *p-val* (95%CI)		
	Model 1	Model 2	Model 3	Model 1	Model 2	Model 3
Gait speed	0.121, *p*<.001 (0.061, 0.181)	-	0.100, *p*=.001 (0.041, 0.16)	0.015, *p*=.05 (4.1E-6, 0.03)	-	0.007, *p*=.34 (-0.007, 0.021)
PC #1	-	0.217, *p*<.001 (0.130, 0.305)	0.194, *p*<.001 (0.107, 0.282)	-	0.078, *p*<.001 (0.056, 0.101)	0.077, *p*=<.001 (0.054, 0.099)
Explained variance	4%	6%	9%	<1%	15%	15%
